# Human Regulatory T Cells of G-CSF Mobilized Allogeneic Stem Cell Donors Qualify for Clinical Application

**DOI:** 10.1371/journal.pone.0051644

**Published:** 2012-12-12

**Authors:** Sya N. Ukena, Sarvari Velaga, Lilia Goudeva, Philipp Ivanyi, Sven Olek, Christine S. Falk, Arnold Ganser, Anke Franzke

**Affiliations:** 1 Department of Hematology, Hemostasis, Oncology and Stem Cell Transplantation, Hannover Medical School, Hannover, Germany; 2 Institute for Transfusion Medicine, Hannover Medical School, Hannover, Germany; 3 Institute for Transplant Immunology, IFB-Tx, Hannover Medical School, Hannover, Germany; 4 Epiontis GmbH, Berlin, Germany; Purdue University, United States of America

## Abstract

Recent clinical studies demonstrate the high potency of regulatory T cells (Tregs) to control graft-versus-host disease in hematopoietic stem cell transplantation (SCT). However, the adoptive transfer of Tregs is limited by their low frequency in unstimulated donors and considerable concerns that G-CSF induced SC mobilization might have negative effects on the stability and function of Tregs. The isolation of Tregs from the G-CSF mobilized SC grafts would extend this novel strategy for tolerance induction to the unrelated setting and simplify global clinical application. We characterized CD4^+^CD25^high^CD127^−^ Tregs from SC donors before and after G-CSF mobilization for their phenotype, function, and stability. After G-CSF application the Treg cell yield increased significantly. Donor Tregs retained their cytokine profile, phenotypic characteristics and *in vitro* expansion capacity after SC mobilization. Most importantly, *in vivo* G-CSF stimulated Tregs remained highly suppressive on the proliferation of effector T cells, also after *in vitro* expansion, and displayed a stable phenotype in epigenetic studies. The surface expression of CXCR3 is transiently reduced. However, donor-derived Tregs maintain their migratory properties after G-CSF stimulation. Therefore, the adoptive transfer of Tregs from G-CSF mobilized SC donors seems to be a feasible and safe strategy for clinical application in allogeneic SCT.

## Introduction

Regulatory T cells (Tregs) play a pivotal role in transplantation tolerance, autoimmunity, infectious diseases and cancer. Currently, clinical approaches worldwide aim to maximize the benefits and to overcome the challenges and risks of Treg cell therapy [1]. In stem cell transplantation experimental model systems have clearly shown that adoptive Treg cell transfer prevents graft-versus-host disease (GvHD) while preserving the beneficial graft-versus-leukemia effect [2] and promoting antiviral immunity [3]. First clinical trials of freshly isolated donor Tregs demonstrate their beneficial effects in prevention of acute GvHD [4], [5], improvement of immune reconstitution and immunity against infectious pathogens [5]. However, the translation of adoptive Treg cell transfer strategies for tolerance induction to the clinic is limited so far to the family donor setting as current studies avoid the isolation of Tregs from G-CSF mobilized stem cell grafts. Because of major concerns that G-CSF exerts negative effects on Treg cell phenotype and function, donor Tregs are isolated from additional aphereses before G-CSF stimulation of the donor. Growing evidence indicates that G-CSF effects are not limited to the myeloid lineage [6] but also induce pleiotropic modulations of adaptive immune responses [7]. This may be reflected by the functional expression of the G-CSF receptor in other cell types like T lymphocytes Most importantly, G-CSF induces alterations of cytokine networks [8], [9], polarization of T cell function [10]–[13] and augmentation of IL-10 producing Tregs [14], [15]. Moreover, T cells from donors treated with G-CSF have a reduced capacity to induce GvHD [12] and show a diminished proliferative response of T cells to allogeneic and mitogenic stimulation [16] probably resulting from the induction of Tr1-like regulatory T cells producing high amounts of IL10 and to a lesser extent TGF-β [17]. These observations have led to major concerns that donor Tregs after SC mobilization might display an induced and instable suppressive phenotype functionally differing from naturally occurring donor Tregs before G-CSF stimulation. This is of high relevance for the clinical application of Tregs as an instable phenotype especially in an inflammatory environment like GvHD might implicate a redirection towards effector T cells leading to an exacerbation rather than amelioration of life-threatening allogeneic immune responses. Furthermore, immune homeostasis after allogeneic SCT demands that adoptively transferred donor Tregs should display efficient suppressive capacity, proliferative response and migration potency to secondary lymphoid organs as well as to the target organs of GvHD in order to control allogeneic immune responses efficiently. Therefore, CD4^+^CD25^high^CD127^-^ donor Tregs have been isolated before and after G-CSF mobilization and comparatively analyzed for their stability, suppressive function, phenotypic characteristics, cytokine profile, migration potency, and expansion capacity.

## Materials and Methods

### Donor Sampling

Prior to sample collection approval was given by the institutional ethics committee of Hannover Medical School. After obtaining signed written informed consent forms from 86 stem cell donors peripheral blood withdrawals were taken before (n = 16 female; mean age: 37.6 years; range: 30–50 years and n = 27 male; mean age: 37.8 years; range: 19–53 years) and after G-CSF administration (n = 9 female; mean age: 38.4 years; range: 30–47 years and n = 34 male; mean age: 38.1 years; range: 25–62 years). HSC mobilization was performed by the subcutaneous administration of 10 µg/kg/d G-CSF (filgrastim; Amgen, Thousand Oaks, CA) for 4 consecutive days.

### Treg Cell Isolation for Further Studies

Heparinized blood samples of 40 ml were obtained from stem cell donors before and after G-CSF administration. Peripheral mononucleated cells (PBMCs) were isolated by Ficoll density gradient centrifugation. Tregs were separated in two steps because of massive increase of granulocytes after G-CSF mobilisation. First, separation of CD4^+^ T cells was performed using the CD4 T-cell isolation Kit II and the AutoMACS separating system (Miltenyi Biotec) according to manufacturer’s instructions. Subsequently, CD4^+^ T cells were stained with monoclonal antibodies against CD14 APC (M0P9, BD Pharmingen), CD4 FITC (RPA-T4, BD Pharmingen), CD25 PE (4E3, Miltenyi Biotech) and CD127 Alexa647 (HiL7-R-M21,BD-Pharmingen). CD14^−^CD4^+^CD25^high^ Tregs were isolated by fluorescence activated cell sorting using a FACSAria Cytometer. Isolated target Treg populations were CD127^−^ and displayed a purity >96%.

### Immune Phenotyping of Tregs

Phenotype of Treg cells was determined by direct immunofluorescence with monoclonal antibodies against the antigens with following fluorochromes: CD4 PerCP, CD127 Alexa647, CD45RA PE, Foxp3 Alexa488, CD25 PeCy7, CD8 APC-H7, CD39 APC, CD45RA PE, CD45RO APC, CD31 PE, CD62L PE, ICOS PE, CCR5 PE, CCR7 A647, CD69 APC-Cy7, CXCR3 PE (all BD Pharmingen), LAP APC (R&D Systems), LAG3 FITC (enzo), 4-1BB APC, CD44 PE, Helios PE, GZMA A647 and Ki67 A647 (all Biolegend). In some experiments CCR4 APC (Biolegend) and CXCR4 PE (Biolegend) have been used. Isotype and FMO controls were included for the respective antibodies. Fixation and permeabilization for staining with antibodies against Foxp3 were performed with Foxp3 Buffer set (BD Pharmingen). After surface staining Treg cells were incubated with Fixation Buffer A for 15 min and washed twice. Fixed cells were permeabilized by incubating with Buffer C for 30 min and stained with Foxp3 antibody. Cells were analyzed using FACS Canto (BD) and FACS Diva 6 software.

### Treg Cell Culture and Expansion

Cell culture was performed under aseptic conditions in 96-well u-bottom plates (Greiner bio-one). Isolated Treg cells were cultured in RPMI 1640 containing Glutamax medium (Gibco) supplemented with 10% FCS (Biochrom) for 14 days at 37°C. To avoid contaminations, 50 µg/ml gentamycin (Sigma Aldrich) and 50 µg/ml penicillin/streptomycin (Sigma Aldrich) were added to the medium. Tregs were stimulated by IL-2 500 U/ml (Proleukin S, Novartis) and CD3/CD28 dynabeads (Invitrogen) in a 4∶1 ratio. Dynabeads were changed after seven days and removed after eleven days of culture. Aliquots of supernatants were frozen after overnight culture (d1) and 3, 7, and 14 days *in vitro* expansion and stored at −80°C until analysis. In selected experiments CD4^+^CD25^high^CD127^−^ Tregs were isolated before G-CSF treatment (n = 5) and cultured for 14 days with daily 100 ng/ml G-CSF (Granocyte 13, Chugai) stimulated with CD3/CD28 Dynabeads and 500 U/ml IL2. For overnight culture, freshly cell sorted CD4^+^CD25^high^CD127^−^ Tregs were washed twice with PBS, transferred into a 96-well u-bottom plate and cultured overnight in RPMI 1640 containing Glutamax medium (Gibco) supplemented with 10% FCS (Biochrom), 50 µg/ml gentamycin and 50 µg/ml penicillin/streptomycin at 37°C.

### Phalloidin Assay

Phalloidin assay was performed using a modified protocol described elsewhere (Walcher et al., 2008). Briefly, 5×10^6^ PBMCs were stained with anti human CD4 PerCp (BD Pharmingen) and anti human CD25 PeCy7 (Biolegend) antibodies. Stained cells were washed, resuspended in cell cuture media and prewarmed at 37°C for 30–60 minutes. Subsequently 300 ng IP-10 was added quickly and incubated for 30 seconds. Cells were fixed, permeabilized and stained with anti human Foxp3 A647 antibody (Biolegend) and Phalloidin FITC (Sigma). Cells were analyzed using FACS Canto (BD) and FACS Diva 6 software.

### Cytokine Profiling

Cytokine and chemokine concentrations in supernatants of cultured and expanded CD4^+^CD25^high^CD127^−^ T cells isolated from SC donors before and after G-CSF treatment were quantified by ‘Bio-Plex, Human Cytokine 17-Plex Panel’ and ‘Bio-Plex, TGFβ 3-Plex’ protein arrays according to the manufacturer’s instructions (BioRad Laboratories). A 2-laser array reader (BioRad Laboratories) simultaneously quantifies all cytokines and chemokines of interest. Standard curves and concentrations were calculated with Bio-Plex Manager 4.1.1 on the basis of the 5-parameter logistic plot regression formula. The detection sensitivity of all analyses was between 0.5 and 78000 pg/ml.

### Suppression Assay

CD4^+^CD25^−^ T responder cells (5×10^4^; Tresp) were stained with 0.5 µM CFSE (Invitrogen) for 15 minutes at 37°C according to the manufacturer`s protocol. These CFSE stained Tresp were cultured with 5×10^4^ irradiated (30 Gy) allogeneic PBMCs and autologous Treg cells in the presence of 1 mg/ml purified anti CD3 and anti CD28 antibodies (both from Biolegend) in RPMI 1640 containing Glutamax medium (Gibco) supplemented with 10% FCS (Biochrom), 50 µg/ml gentamycin (Sigma Aldrich) and 50 µg/ml penicillin/streptomycin (Sigma Aldrich). The final suppressor to responder T cell ratios were: 2∶1, 1∶1, 1∶2, 1∶10 and 0∶1 (ctrl). After 7 days, cells were analyzed by FACS Canto (BD). Percentage of suppression was calculated as followed: 100 - (percentage of proliferated Tresp x 100/percentage of proliferated Tresp). For suppression assays after *in vitro* expansion frozen autologous PBMCs were thawed one day before usage and kept under cell culture conditions overnight. CD4^+^CD25^−^ T cells were isolated from thawed autologous PBMCs by depletion of CD4^−^ T cells and positive selection for CD25^+^ T cells using the CD4^+^CD25^+^ Regulatory T Cell *Isolation* Kit (Miltenyi Biotech) and stained with 0.5 µM CFSE (Invitrogen) as described for freshly isolated Tresp.

### DNA Demethylation Analysis of the FOXP3 Gene Locus

Genomic DNA was isolated from >150.000 freshly sorted or expanded regulatory T cells using the AllPrep DNA/RNA Micro Kit (Qiagen) according to manufacturer’s instructions. Concentrations of genomic DNA were measured by Nano Drop (Thermoscientific). DNA demethylation analysis of *foxp3* was carried out as previously described [18]. Results from female donors were corrected with a factor of 2, because one of the Treg specific demethylated region (TSDR) alleles is methylated as a result of X-inactivation.

### Statistical Analysis

GraphPad Prism software has been used for interpretation of data and statistical analysis. For the analyses two-tailed paired Students’ t-test and two-tailed Mann-Whitney t-test were performed. P values with p>0.05 were indicated as not significant (n.s.) and significant p values with p<0.05 as *, p<0.01 as **, p<0.001 as *** and p<0.0001 as ****.

## Results

### G-CSF Application Increases Donor Treg Cell Number

**Figure 1:**
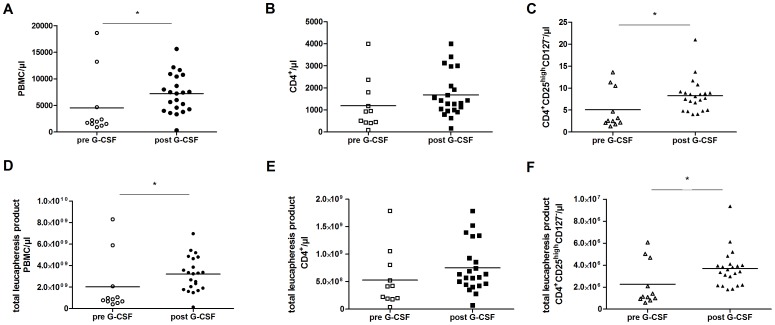
Increase of CD4^+^CD25^high^CD127^−^ Treg cell numbers following G-CSF mobilization. Cell numbers of PBMCs isolated by Ficoll density gradient (A) MACS isolated CD4^+^ T cells (B) and subsequently FACS isolated CD4^+^CD25^high^CD127- Tregs (C) per µl blood withdrawal volume before (n = 11, pre) and after (n = 22, post) G-CSF mobilization. Cell numbers of PBMCs isolated by Ficoll density gradient (D) MACS isolated CD4+ T cells (E) and subsequently FACS isolated CD4^+^CD25^high^CD127^−^ Tregs (F) per µl of the total leukapheresis product. Black lines indicate median values.

Enumeration of cell numbers after isolation processes (Ficoll, MACS and FACS based cell sorting) and in the leukapheresis products revealed significantly increased numbers of PBMCs after G-CSF mobilization (Fig. 1A and D). Although cell numbers of CD4^+^ T cells were not significantly increased in the peripheral blood of the stem cell donors and leukapheresis products (Fig. 1 B and E, respectively), CD4^+^CD25^high^CD127^−^ Tregs increased 1.6 fold after G-CSF mobilization (Fig. 1 C and F, respectively).

### Phenotype Stability and Suppressive Capacity of Donor Tregs

**Figure 2:**
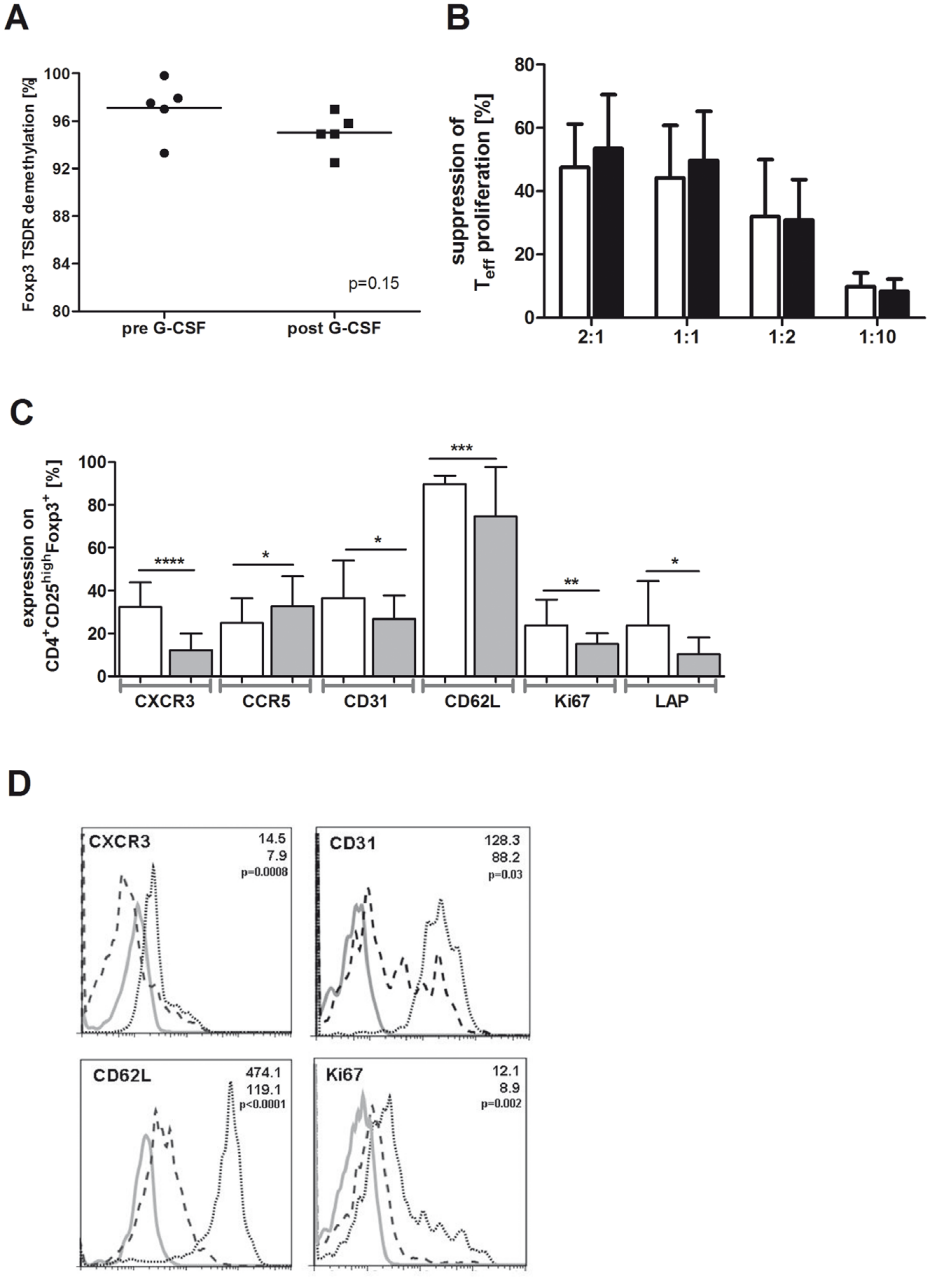
Analysis of donor Tregs before and after SC mobilization: Stability, suppressive function and phenotype. (A) Quantification of the TSDR demethylation levels of the *foxp3* promoter in freshly FACS isolated CD4^+^CD25^high^CD127^−^ Tregs before (pre, n = 5) and after (post, n = 5) G-CSF mobilization. (B) Suppressive capacity of freshly FACS isolated CD4^+^CD25^high^CD127^−^ Tregs before (pre, n = 5, white bars) and after (post, n = 5, black bars) G-CSF mobilization on the proliferation of autologous CD4^+^CD25^−^ T cells stimulated with anti-CD3 and anti-CD28 in the presence of irradiated allogeneic PBMCs. Ratio of Treg cells to CD4^+^CD25^−^ T effector cells is 2∶1, 1∶1, 1∶2 and 1∶10. Percentages of suppression were calculated as indicated in Material and Methods and presented as mean values with standard deviation (SD). (C) PBMCs were isolated before (pre, n = 25, white bars) and after (post, n = 21, grey bars) G-CSF induced stem cell mobilization for FACS analyses. Expression values were calculated as percentage of CD4^+^CD25^high^CD127^−^Foxp3^+^ Tregs and are presented as mean values with SD (error bars). (D) In addition mean fluorescence intensities (MFI) were determined and representative overlays from histogram plots are shown for candidates with significant expression differences. Spotted black lines represent pre G-CSF and dashed black lines indicate post G-CSF. Grey lines show the respective isotype control.

The demethylated status of the *Foxp3* locus (TSDR) specifies the committed state of regulatory T cells [19]. As shown by the comparative quantification of the TSDR demethylation, G-CSF application does not affect the stability of the Treg cell phenotype in stem cell donors (mean 97.1%, range 93.3–99.8% pre G-CSF vs mean 95% range 92.5–97% post G-CSF; p = 0.15; Fig. 2A). Accordingly, the suppressive capacity of CD4^+^CD25^high^CD127^−^ Tregs after stem cell mobilization is comparable to Tregs before G-CSF stimulation of the stem cell donors (Fig. 2B). Although not significant, Tregs tend to be more suppressive at the timepoint of stem cell apheresis (53.5% ±17 vs 47.6% ±13.8 at a 2∶1 ratio and 49.8% ±15.4 vs 44.2% ±16.5 at a 1∶1 ratio).

### Phenotypic Characterization of Donor Tregs

To clarify whether G-CSF induces changes of the CD4^+^CD25^high^CD127^−^ Treg cell phenotype, we performed a comprehensive FACS analysis (see Material and Methods). Significant changes following G-CSF stimulation of the stem cell donors could be observed. Compared to the protein expression before G-CSF stimulation the chemokine receptor CXCR3 (mean 32.3% vs. mean 12.2%), the thymic marker CD31 (mean 36.6% vs. mean 26.7%), the activation marker CD62L (mean 89.7% vs. mean 74.7%), the proliferation marker Ki67 (mean 23.8% vs. mean 15.1%) and TGFβ inhibiting protein LAP (mean 23.8% vs. mean 10.3%) were significantly reduced in Tregs at the timepoint of leukapheresis, whereas CCR5 surface expression (mean 24.8 vs. mean 32.7) was induced (Fig. 2C). Moreover, the density of surface expression presented as mean fluorescence intensities (MFI) of CXCR3, CD62L and Ki67 were also significantly decreased after G-CSF application (Fig. 2D). Interestingly, no significant changes were obtained in CD4^+^CD25^high^CD127^−^Foxp3^+^ Tregs for the expression of the activation markers CD69 and CD44, costimulatory molecule ICOS, CD45RA and CD45RO, Treg cell markers CD127, HELIOS, 4-1BB, and CD39, migratory receptor CCR7, CXCR4, CCR4 and functionally relevant molecules like LAG3, GZMA, and IL-10 (Table 1).

**Table 1:** Phenotype of donor Tregs before (pre) and after (post) G-CSF mobilization.

CD4^+^CD25^high^Foxp3^+^	preG-CSF	SD	postG-CSF	SD	p value
CXCR3^+^	32.3	±11.6	12.2	±6.2	0.00
CCR5^+^	24.8	±11.7	32.7	±14.4	0.04
CD31^+^	36.6	±17.4	26.7	±11.7	0.03
CD62L^+^	89.7	±4.1	74.7	±24.6	0.00
Ki67^+^	23.8	±11.9	15.1	±5.2	0.00
LAP^+^	23.8	±20.7	10.3	±8.1	0.01
CXCR4^+^	87.2	±7.6	79.2	±20.5	0.38
CCR4^+^	61.9	±10.7	59.9	±14.1	0.77
CCR7^+^	44	±22.3	50	±21.1	0.37
ICOS^+^	26.3	±15.7	22.8	±19.6	0.49
CD39^+^	40.8	±25.1	43.6	±19.6	0.68
CD127^+^	5	±5.5	6.1	±5.2	0.47
CD45RA^+^	24	±17.4	33.4	±16.6	0.07
CD45RO^+^	74.5	±20.0	70.5	±13.7	0.43
CD69^+^	15.7	±15.2	9.5	±4.8	0.08
CD44^+^	95.7	±20.8	95.2	±23.5	0.95
GRZMA^+^	5.7	±6.8	7.4	±18.3	0.65
4-1BB^+^	13.1	±11.1	14.4	±13.4	0.73
HELIOS^+^	40.2	±11.4	44.1	±13.0	0.31
LAG3^+^	12.6	±13.9	14.1	±13.3	0.73
IL10^+^	0.8	±1.3	1	±0.8	0.72

PBMCs were isolated before (pre, n = 25) and after (post, n = 21) G-CSF induced stem cell mobilization for FACS analyses. CXCR4 and CCR4 expression was determined on PBMCs isolated from n = 6 before and n = 7 SC donors after G-CSF induced SC mobilization. Expression values were calculated as percentage of CD4^+^CD25^high^CD127^−^Foxp3^+^ Tregs and are presented as mean values with standard deviation (SD). p values were calculated with two-tailed Student’s ttest.

It is well known that G-CSF exerts direct and indirect effects on T cells [20]. To test whether *in vivo* G-CSF mediated effects on the Treg cell phenotype are directly inducible, Tregs were isolated from donors before G-CSF mobilization and stimulated *in vitro* with G-CSF. Phenotypic markers of donor Tregs showing a significant change of protein expression after G-CSF induced SC mobilization (Table 1, Fig. 2C) were analyzed before and after G-CSF stimulation *in vitro*. In contrast to G-CSF stimulation *in vivo* direct stimulation of Tregs with G-CSF did not significantly affect the protein expression of CXCR3, CCR5, CD31, CD62L, Ki67 and LAP (Fig. S1).

### Functional Analysis of Th1 Associated Migratory Capacity of Donor Tregs

**Figure 3:**
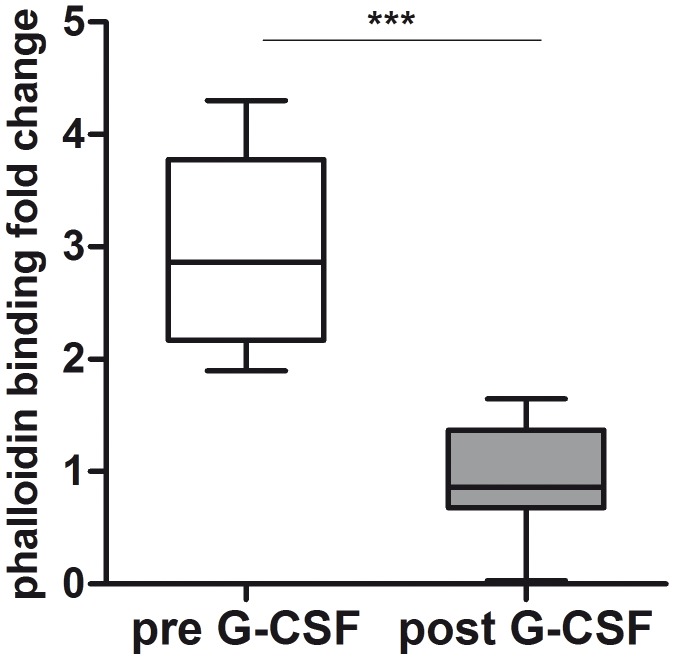
Th1 associated migration capacity of donor Tregs before and after SC mobilization. PBMCs isolated from stem cell donors before (n = 6) and after (n = 13) SC mobilization were stained with anti human CD4 and CD25 antibodies and pretreated with 300 ng IP10, the ligand of CXCR3. Subsequently cells were stained with anti human Foxp3 A647 and Phalloidin FITC in order to measure induction of F-actin polymerization as a consequence of stimulation with the corresponding ligand. Data are shown as fold change of Phalloidin detection and presented as box whisker plots.

The decreased surface expression of the Th1 associated migratory receptor CXCR3 after G-CSF application might have negative functional effects on the migratory potency of donor Tregs. Migration in response to chemotactic stimuli requires a complex rearrangement of the cell actin cytoskeleton. Initial step is the extension of the cell membrane through controlled actin polymerization. Chemokine-induced induction of actin polymerization can be measured via fluorescent phalloidin that binds with a high affinity to stabilized F-actin filaments. Freshly isolated Tregs after G-CSF administration showed significantly decreased levels of F-actin after stimulation with IP10 as ligand of CXCR3 (Fig. 3) detected by phalloidin assay. Compared to donor Tregs before G-CSF stimulation, the levels of F-actin decreased in mean 2.97 fold in response to IP10 following G-CSF *in vivo*.

**Figure 4:**
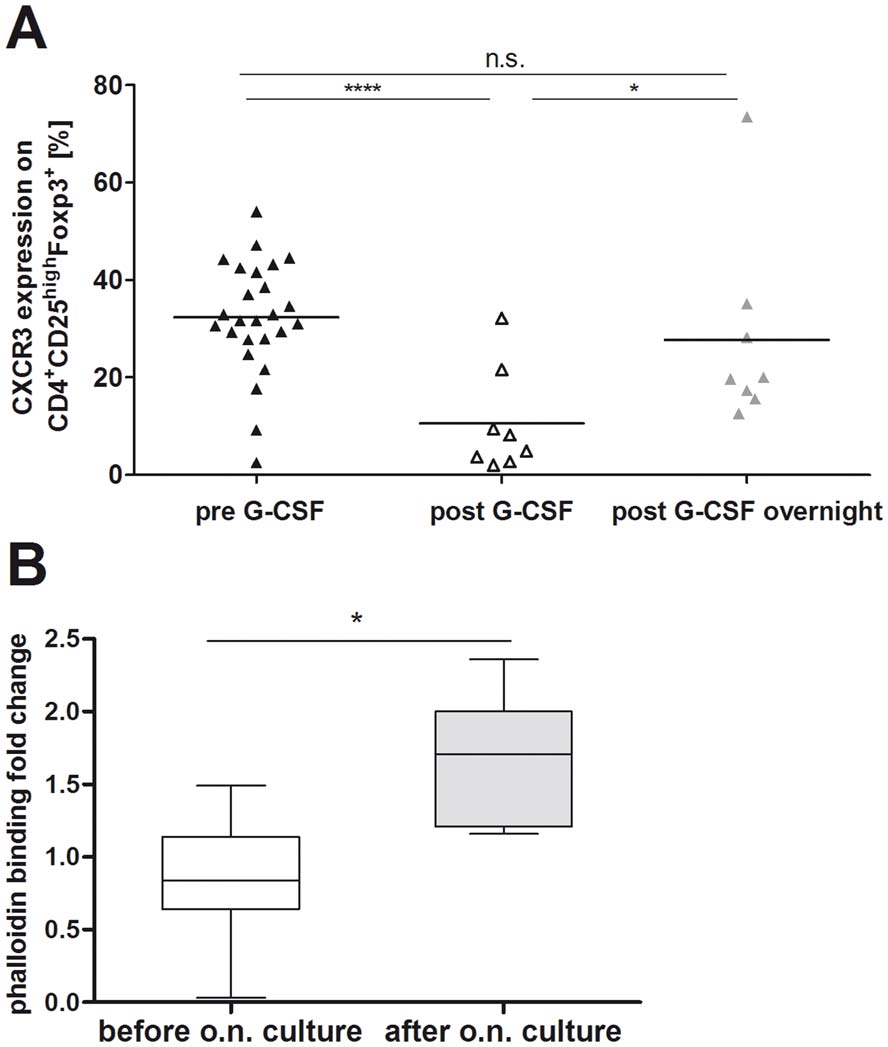
Donor Tregs maintain their migratory potency after SC mobilization. Freshly isolated PBMCs were cultured overnight in cell culture media. After 12 hours of *ex vivo* culture, PBMCs were reanalyzed by FACS for CXCR3 surface protein expression (A) and F-actin accumulation after challenging with the corresponding chemokine ligand IP10 (B). CXCR3 expression is shown as percentage of CD4^+^CD25^high^CD127^−^Foxp3^+^ regulatory T cells analyzed before (black triangles) and after (white triangles) G-CSF *in vivo* mobilization as well as after overnight culture (grey triangles). Black lines indicate mean values (A).The phalloidin binding to F-actin following 30 seconds stimulation with 300 ng IP10 is shown as fold change of Phalloidin detection and presented as box whisker plots.

In order to test whether donor Tregs retain their initial surface expression of the migratory chemokine receptor CXCR3, donor PBMCs were isolated after G-CSF induced stem cell mobilization and cultured overnight under standard cell culture conditions. In comparison to freshly isolated CD4^+^CD25^high^Foxp3^+^ Tregs at the timepoint of leukapheresis, CXCR3 protein expression on donor Tregs increased significantly (p = 0.04) after overnight culture (Fig. 4A) and reached the initial protein expression levels before G-CSF treatment of the donors. The increased surface expression of CXCR3 correlated with the functional recovery of donor Tregs tested by the phalloidin assay. The incorporation of phalloidin in response to the CXCR3 ligand IP10 in donor Tregs increased significantly after overnight culture (mean 0.8 vs. 1.8%; Fig. 4B). Therefore, G-CSF induced reduction of the Th1 associated chemokine receptor CXCR3 occurs only transiently and donor Tregs maintain their migratory capacity.

### Cytokine Profile, Proliferative and Suppressive Capacity of Expanded Donor Tregs

**Figure 5:**
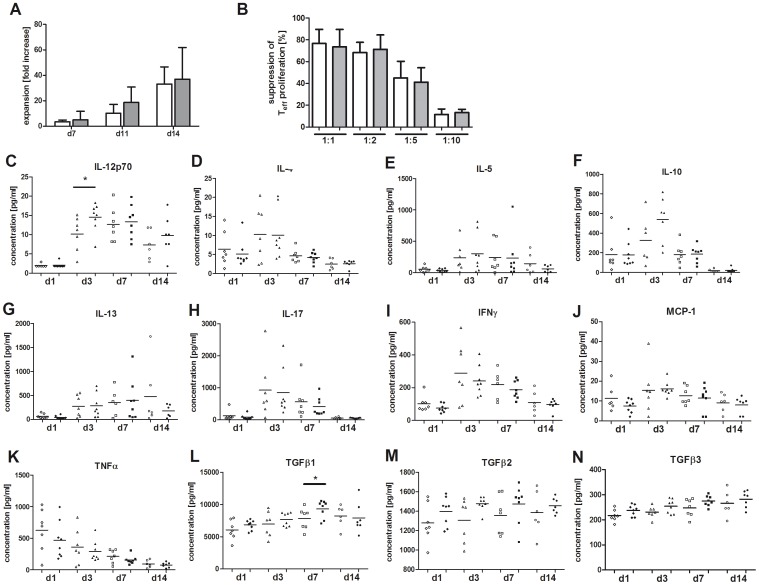
Analysis of donor Tregs before and after SC mobilization: Expansion capacity and cytokine profile. (A) Expansion capacity is shown as increase of cell counts with fold change after 7, 11 and 14 days of *in vitro* stimulation with anti-CD3/−CD28 Dynabeads and 500 U/ml IL2. Data are presented for CD4^+^CD25^high^CD127^−^ Tregs isolated before (n = 8; white bars) and after (n = 11; grey bars) G-CSF mobilization as mean values. Error bars represent SD. (B) Suppressive capacity of *in vitro* expanded CD4^+^CD25^high^CD127^−^ Tregs before (pre, n = 4, white bars) and after (post, n = 4, grey bars) G-CSF induced SC mobilization on the proliferation of autologous CD4^+^CD25^−^ T cells. Ratio of Treg cells to CD4^+^CD25^−^ T effector cells is 1∶1, 1∶2 and 1∶10. Percentages of suppression were calculated as indicated in Material and Methods and presented as mean values with standard deviation (SD). (C–N) Supernatants from *ex vivo* cultured CD4^+^CD25^high^CD127^−^ Tregs (open symbols: before G-CSF application; closed symbols: after G-CSF application) were collected on day 1 (diamonds), 3 (triangles), 7 (squares) and 14 (circles) and comparatively analyzed for the secretion of Th1, Th2, pro – and anti-inflammatory cytokines (see Material and Methods).

With respect to future clinical trials the use of *in vitro* expanded Tregs might be advantageous aiming at higher Treg cell numbers or manufacturing of antigen-specific Tregs. Therefore, freshly isolated CD4^+^CD25^high^CD127^−^ donor Tregs were expanded for 14 days in order to test whether G-CSF application in stem cell donors influences their expansion capacity, suppressive potential or cytokine profile. The *in vitro* expansion of Tregs from G-CSF mobilized donors resulted in higher cell numbers increasing up to 88.2-fold (mean 36.9-fold) compared to 52-fold (mean 33-fold) before G-CSF mobilization (Fig. 5A). The proliferative capacity of Tregs after G-CSF application did not significantly increase but could be maintained in comparison to unstimulated donors. Functional assays revealed that G-CSF application is also not reducing the suppressive capacity of *in vitro* expanded donor Tregs. *In vitro* expanded donor Tregs remain highly suppressive on the proliferation of effector T cells before and after G-CSF induced SC mobilization (Fig. 5B). *In vitro* stimulated Tregs were additionally compared for their cytokine profile in order to characterize possible effects of G-CSF stimulation *in vivo* on the Treg cell phenotype (Fig. 5 C–N). Freshly isolated Tregs (day 1 before start of *in vitro* stimulation) showed a spontaneous secretion of IL-10, IL-17, IFNγ, TNFα and TGFβ1,-2 and -3. However, no significant difference could be observed for cytokine secretion by Tregs before and after G-CSF application of the SC donors. After 3 days of *ex vivo* culture an increase of cytokine production could be observed for IL-10, IL-4, IL-5, IL-12p70, IL-13, IL-17, IFNγ, and MCP1, while the concentration of TNFα decreased. However, significant differences between donor Tregs before and after G-CSF mobilization were only detected at day 3 for IL-12p70 (p<0.05; Fig. 5C) and after one week for TGFβ1 (p<0.05; Fig. 5L) with higher secretion levels after G-CSF application of the SC donors. Concentrations of IL-4, IL-5, IL-13, IL-17, IFNγ, MCP1, TNFα, TGFβ2, and TGFβ3 did not differ significantly between expanded donor Tregs before and after *in vivo* G-CSF stimulation.

### Phenotypic Characterization of Donor Tregs after *in vitro* Expansion

**Figure 6:**
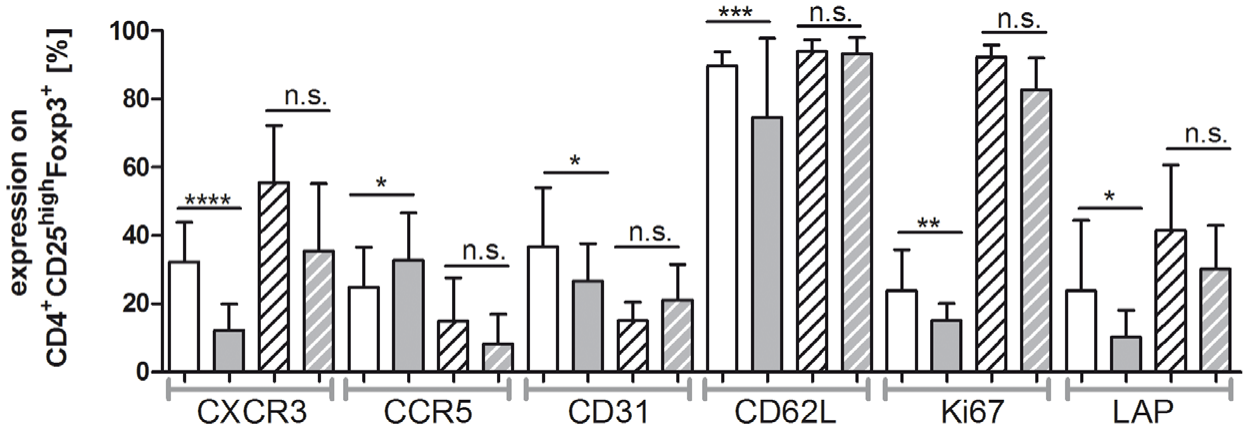
Phenotypic analysis of *in vitro* expanded donor Tregs before and after SC mobilization. PBMCs were isolated before (n = 25) and after (n = 21) G-CSF mobilization. FACS isolated CD4^+^CD25^high^CD127^−^Foxp3^+^ donor Tregs were expanded *ex vivo* for 14 days. FACS analysis was performed before (white bars) and after (grey bars) SC mobilization as well as after *in vitro* expansion of donor Tregs isolated before (white with black stripes) and after (grey with white stripes) SC mobilization. Expression values were calculated as percentage of CD4^+^CD25^high^CD127^−^Foxp3^+^ Tregs and their mean values are presented as bar graphs with SD as error bars.

The phenotypic analysis of donor Tregs after 14 days of *in vitro* culture was comparatively performed for Tregs before and after G-CSF stimulation of the stem cell donors (see Material and Methods for overview on the analyzed phenotypic marker). Most importantl*y,* G-CSF stimulation of the stem cell donors does not lead to significant alterations of the phenotypic profile of donor Tregs after 14 days of *in vitro* expansion (Fig. 6). Thus, G-CSF induced significant changes of the analyzed phenotypic marker at the timepoint of leukapheresis (CXCR3, CCR5, CD31, CD62L, Ki67, LAP) reverted after 14 days *in vitro* expansion of donor Tregs. Notably, the G-CSF induced reduction of CXCR3 surface expression, which recovered after overnight culturing of donor Tregs, remained at significantly higher level after 14 days *in vitro* expansion.

## Discussion

Current clinical trials of adoptive Treg cell transfer for the prevention of GvHD following allogeneic SCT circumvent the isolation of donor Tregs after G-CSF induced SC mobilization. Accumulating evidence indicates that G-CSF effects are not limited to the myeloid lineage [6] but also induce pleiotropic modulations of adaptive immune responses [7]. Most importantly, G-CSF induces alterations of cytokine networks [8], [9], polarization of T cell function [10]–[13] and augmentation of IL-10 producing Tregs [14], [15]. This raises major concerns that donor Tregs after SC mobilization might display an induced and instable suppressive phenotype functionally differing from naturally occurring donor Tregs before G-CSF stimulation. However, the isolation of donor Tregs from the G-CSF stimulated SC apheresis would simplify adoptive Treg cell transfer strategies and allow the global clinical application of donor Tregs also in the unrelated transplant setting (Fig. S2). Therefore, CD4^+^CD25^high^CD127^−^ donor Tregs were isolated before and after G-CSF mobilization and comparatively analyzed for their stability, suppressive function, phenotypic characteristics, cytokine profile, migration potency and expansion capacity.

G-CSF application leads to a numerical increase of circulating donor Tregs with a significant higher Treg cell yield in the SC graft, while the number of CD4^+^ T cells is not significantly affected. CXCR4 expressing donor Tregs retain their predominant central memory phenotype (CD45RA^+^CD62L^hi/int^) and do not show signs of activation/proliferation arguing for homeostatic trafficking from the bone marrow (BM) as natural reservoir of Tregs to the peripheral blood [21]. Similar to the mobilization of hematopoietic stem cells (HSC) interaction of CXCR4 with stromal derived factor 1 (SDF1/CXCL12) modulated by G-CSF stimulation generates signals not only regulating the trafficking of hematopoietic SC from the BM to peripheral blood [22], [23] but also of T cell populations [24], [25]. During G-CSF induced HSC mobilization, CXCL12 protein expression in the bone marrow decreases, and CXCR4 expressing HSC and Tregs can be consequently released from bone marrow. Our data show a high and stable expression of CXCR4 on Tregs before and after G-CSF application. Furthermore, the lacking upregulation of the proliferation marker Ki67 argues against G-CSF induced expansion of donor Tregs in the periphery. In addition, G-CSF treatment led to a significant reduction of CD31 on donor Tregs which is known to be expressed on recent thymic emigrants [26]. The higher Treg cell yield in the SC graft facilitates sufficient isolation of this rare cell population for clinical applications.

The stability of the suppressive phenotype is of high relevance for the validity and safety of human Treg cell therapy as spontaneous or environmentally induced reprogramming of Foxp3^+^ Tregs into effector T cells would be detrimental. Very recently, Miyao et al. could demonstrate that Treg cells constitute a stable cell lineage the committed state of which is ensured by DNA demethylation of the *FoxP3* locus (TSDR) also in an inflammatory environment [19]. The quantification of the demethylated status of the TSDR indicates that CD4^+^CD25^high^CD127^-^ donor Tregs after G-CSF stimulation represent a stable cell lineage robustly maintaining their committed state also in a changing environment. Moreover, donor Tregs remain highly suppressive on the proliferation of effector T cells correlating with a stable expression of relevant molecules like LAG3, CD39, GZMA and IL-10 following G-CSF stimulation [27]. After G-CSF stimulation donor Tregs show a significantly reduced surface expression of the TGFß binding protein LAP correlating with higher TGFß secretion of *in vitro* expanded post G-CSF donor Tregs and enhancing their suppressive capacity. With respect to adoptive Treg cell transfer strategies for the prevention and therapy of GvHD, a stable and highly suppressive phenotype of donor Tregs is of pivotal relevance and might compensate the reduced suppressive capacity of Tregs in GvHD patients recently reported by us [28]. Furthermore, a significant proportion of the long-term CD44^hi^CD62L^+^CD69^−^ memory Tregs shows a cleavage of CD62L after G-CSF application. However, no signs of activation or induction of the co-stimulatory molecules ICOS or 4-1BB could be observed in donor Tregs. As terminally differentiated activated CD45RA^−^Foxp3^hi^ Tregs rapidly die after TCR stimulation *in vitro* and *in vivo* [29], the resting Treg cell phenotype observed after G-CSF stimulation is favourable for the clinical application of donor Tregs. Notably, the protein expression of the transcription factor HELIOS as marker for naturally occurring Tregs [30] is not modified by G-CSF stimulation *in vivo*.

The migration of Tregs to secondary lymphoid organs and target organs of GvHD is critical for effective control of allogeneic immune responses and immune homeostasis following allogeneic SCT. Recently, we have demonstrated that Tregs of GvHD patients show a significant reduction of CCR5 and CXCR3 surface expression likely leading to a diminished migration capacity of Tregs with significantly lower Treg cell infiltration in the inflamed intestinal mucosa [28]. Whereas G-CSF stimulation of the SC donors induces CCR5 surface expression on donor Tregs, the Th1 associated migratory receptor CXCR3 is transiently reduced. However, Tregs of the SC donors completely retain their CXCR3 surface expression level and migratory capacity in response to the respective ligand IP10. Therefore, Tregs of G-CSF stimulated SC donors exhibit a beneficial migratory receptor profile likely restoring potential functional defects of Tregs in patients developing a GvHD. Moreover, expression of the skin homing receptor CCR4 [31] is also not affected by G-CSF probably indicating that Tregs of SC donors exhibit the capability to migrate to inflammatory sites in the context of skin GvHD.

The comparative analysis of the Treg cytokine profile reveals no differences in the spontaneous secretion whereas *in vitro* stimulation of Tregs after SC mobilization induces the release of higher levels of IL-12 and TGFß1. However, the secretion of other key cytokines (like IL-10, IFN-y, TNFa, IL-17, IL-2, IL-4) appears to be independent of G-CSF stimulation and is comparable to data from other investigators [32]. Notably, IFNγ and other cytokines associated with effector functions could be coexpressed in Tregs and are not mutually exclusive [33]. Interestingly, *in vitro* stimulation of Tregs isolated after SC mobilization aligned the expression profile of donor Tregs before and after G-CSF stimulation. Current research focuses on the development of large-scale expansion protocols for Tregs with higher cell yields [32], [34]–[36]. The expansion capacity of donor Tregs after G-CSF stimulation is higher than before SC mobilization underlining their proliferative potency with maintained suppressive capacity.

In conclusion, our data indicate donor Tregs after G-CSF stimulated SC mobilization retain their potent suppressive and stable phenotype combined with a favorable migration capacity and attractive cell yield. This study outlines that the adoptive transfer of donor Tregs after G-CSF stimulation appears to be feasible and safe. The isolation of donor Tregs from the SC graft will simplify their global clinical application and extend this novel strategy of tolerance induction to the unrelated transplant setting.

## Supporting Information

Figure S1 **In vitro effects of G-CSF on Tregs.** CD4^+^CD25^high^CD127^−^Foxp3^+^ Tregs were isolated before G-CSF treatment (n = 5) and cultured for 14 days with (black bars) and without (white bars) 100 ng/ml G-CSF stimulated with CD3/CD28 Dynabeads and 500 U/ml IL2. Percentage of CXCR3, CCR5, CD31, CD62L, Ki67 and LAP protein expression of CD4^+^CD25^high^CD127^−^ Tregs is shown. For statistical analysis two-tailed Mann Whitney t-test and unpaired t-test was performed, respectively. Not significant p values are indicated as n.s. P values p<0.05 are indicated as *, p<0.01 as **, p<0.001 as *** and p<0.0001 as ****.
(TIF)

Figure S2 **Schematic presentation of protocols for adoptive Treg cell transfer.** (A) Currently used protocols for clinical trials include isolation of Tregs from the apheresis product befpre G-CSF mobilization of the stem cell donor and injection into the recipient at day −4. A second apheresis is necessary at d0 to isolate the graft. Consequently this protocol is only feasible in a related donor setting. (B) An additional apheresis is not necessary if Tregs are isolated after G-CSF application. This setting is feasible for related and unrelated donors. Moreover, neutropenia is not prolongated.
(TIF)
