# Supplementary figures and images for: Human Regulatory T Cells of G-CSF Mobilized Allogeneic Stem Cell Donors Qualify for Clinical Application
Source: PLoS One. 2012 Dec 12;7(12):e51644. doi: 10.1371/journal.pone.0051644 (PMC3520921; doi:10.1371/journal.pone.0051644)

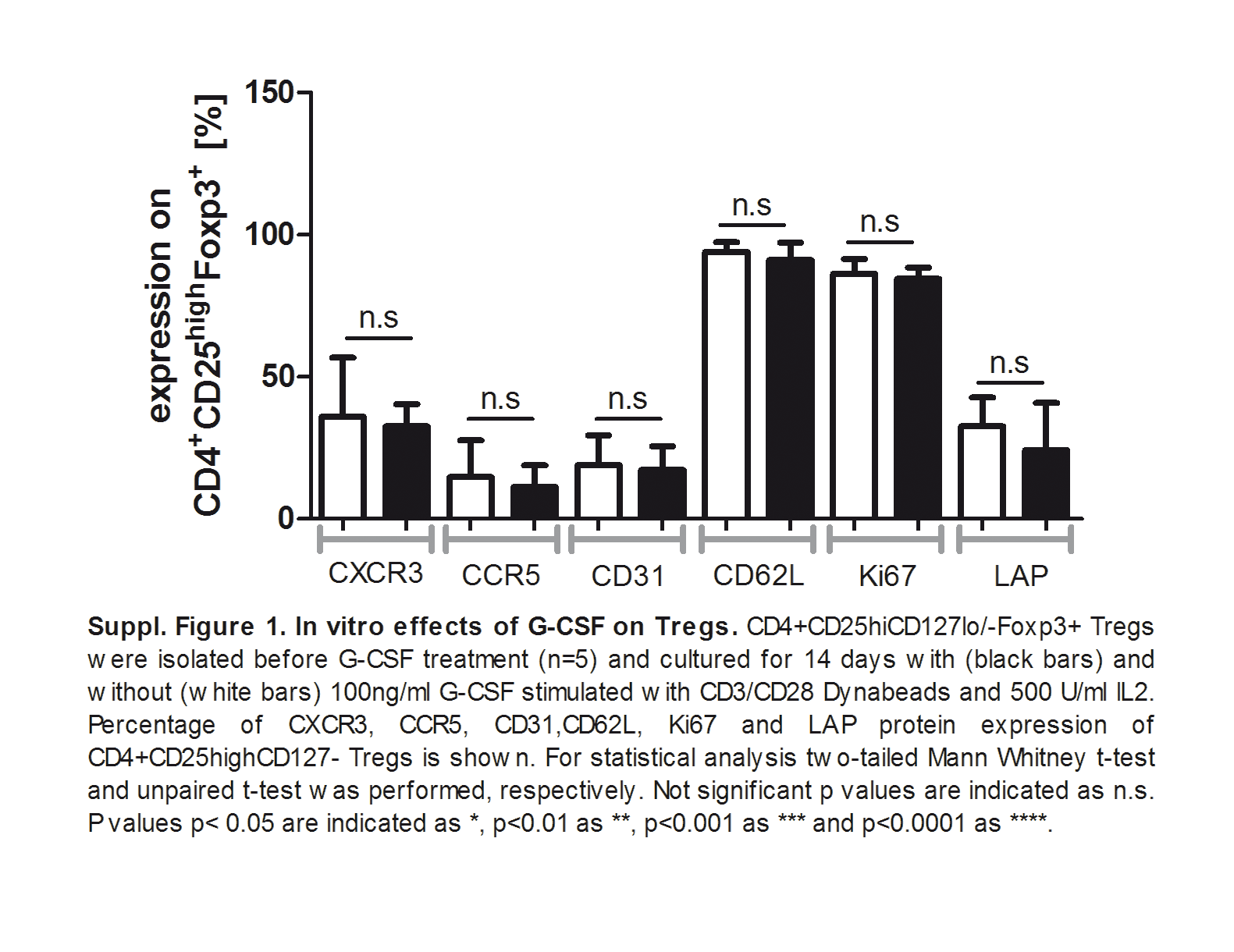

Supplement: Figure S1 — In vitro effects of G-CSF on Tregs. CD4+CD25highCD127−Foxp3+ Tregs were isolated before G-CSF treatment (n = 5) and cultured for 14 days with (black bars) and without (white bars) 100 ng/ml G-CSF stimulated with CD3/CD28 Dynabeads and 500 U/ml IL2. Percentage of CXCR3, CCR5, CD31, CD62L, Ki67 and LAP protein expression of CD4+CD25highCD127− Tregs is shown. For statistical analysis two-tailed Mann Whitney t-test and unpaired t-test was performed, respectively. Not significant p values are indicated as n.s. P values p<0.05 are indicated as *, p<0.01 as **, p<0.001 as *** and p<0.0001 as ****. (TIF) [file pone.0051644.s001.tif]

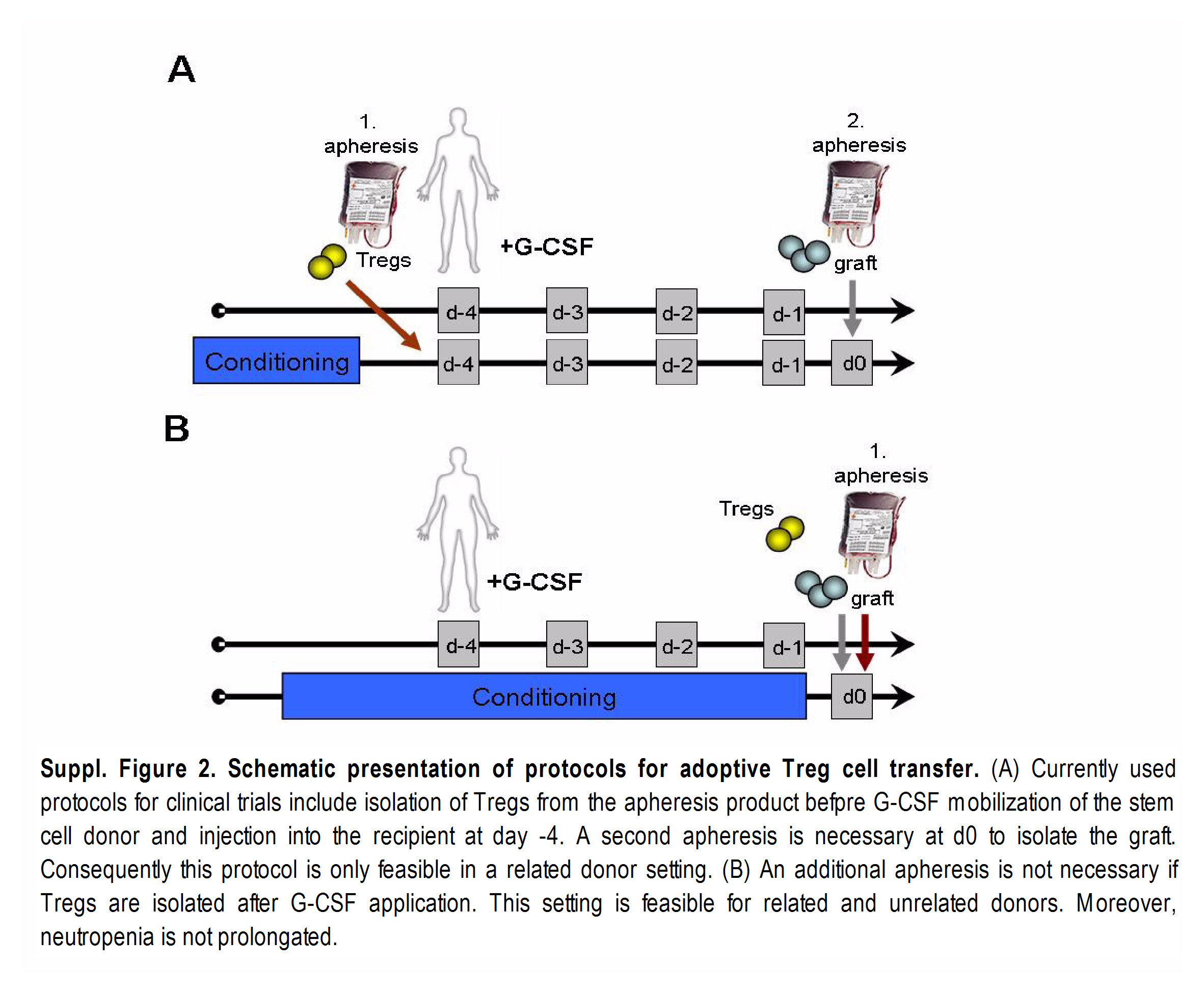

Supplement: Figure S2 — Schematic presentation of protocols for adoptive Treg cell transfer. (A) Currently used protocols for clinical trials include isolation of Tregs from the apheresis product befpre G-CSF mobilization of the stem cell donor and injection into the recipient at day −4. A second apheresis is necessary at d0 to isolate the graft. Consequently this protocol is only feasible in a related donor setting. (B) An additional apheresis is not necessary if Tregs are isolated after G-CSF application. This setting is feasible for related and unrelated donors. Moreover, neutropenia is not prolongated. (TIF) [file pone.0051644.s002.tif]
